# Complete Genome Sequence of *Bordetella pertussis* D420

**DOI:** 10.1128/genomeA.00657-15

**Published:** 2015-06-11

**Authors:** Christine J. Boinett, Simon R. Harris, Gemma C. Langridge, Elizabeth A. Trainor, Tod J. Merkel, Julian Parkhill

**Affiliations:** a Wellcome Trust Sanger Institute, Hinxton, Cambridgeshire, United Kingdom; b Division of Bacterial, Parasitic and Allergenic Products, Center for Biologics Evaluation and Research, FDA, Bethesda, Maryland, USA

## Abstract

*Bordetella pertussis* is the causative agent of whooping cough, a highly contagious, acute respiratory illness that has seen resurgence despite the use of vaccines. We present the complete genome sequence of a clinical strain of *B. pertussis*, D420, which is representative of a currently circulating clade of this pathogen.

## GENOME ANNOUNCEMENT

Whooping cough is a highly contagious acute respiratory infection caused by the Gram-negative pathogen *Bordetella pertussis*, whose spread is thought to be through aerosolized respiratory droplets (1). Despite vaccination efforts that began in the 1940s, reported cases of pertussis are increasing, with more than 28,000 in 2014 in the United States (CDC: http://www.cdc.gov/pertussis/surv-reporting.html) and an estimated 16 million worldwide (WHO: http://www.who.int/wer/2010/wer8540.pdf). The reason for the increasing incidence in pertussis remains elusive, largely due to gaps in our knowledge of the disease and of how the vaccine works against currently circulating strains. In an effort to shed light on the disease, scientists have used a variety of models to mirror the disease progression in humans, but these have not been successful at producing the full spectrum of disease (2). Using a recent clinical *B. pertussis* isolate, D420 (also known as BPD420), Warfel and colleagues developed a successful model using nonhuman primates (3). We have generated the complete genome sequence of this clinically relevant strain, which is representative of the recent expansion of *ptxP3* and *fim3-2* isolates of *B. pertussis* (4), which will aid our understanding of this disease and strengthen the applicability and interpretation of this animal model.

Genomic DNA from *B. pertussis* strain D420 was extracted and sequenced on the Pacific Biosciences RSII sequencer. The DNA was sheared with the Diagenode Megaruptur to 30 kb and 3.9 µg of sheared DNA was used in library preparation according to the manufacturer’s protocol. We used the P4 polymerase to make the complex and C2 chemistry to yield a total of 91,723 reads with an *N*_50_ of 5.4 kb (postfiltering), generating 100× coverage of the genome. *De novo* assembly of the resulting reads was performed using HGAP.3 (Pacific Biosciences). The sequence assembled into one complete contig, 4.1 Mb in length and with a G+C content of 67.7%. The genome was annotated with Prokka (5) and the start of the sequence was set at *dnaA*.

The maximum likelihood program RAxML (6) was used to place D420 in a whole-genome single nucleotide polymorphism (SNP) phylogenetic tree with a global collection of *B. pertussis* (4). To identify SNPs, sequence reads were mapped against the Tohama I genome sequence as previously described (7). Strain D420 was included by simulating perfect read pairs of 100 bp from the genome sequence *in silico*. The D420 strain clusters among the other recently circulating *B. pertussis* strains harboring the *ptxP3* and *fim3-2* alleles and is very distant to the Tahoma I strain currently used as a reference in many studies, and for which the first genome was sequenced. The availability of this genome can therefore provide an important resource in understanding the genetic context of the current disease and as a reference for further genomic studies.

### Nucleotide sequence accession number. 

This *B. pertussis* genome has been deposited in the European Nucleotide Archive under the accession number LN849008.
