# Erratum for Boinett et al., Complete Genome Sequence of *Bordetella pertussis* D420

**DOI:** 10.1128/genomeA.00842-15

**Published:** 2015-07-16

**Authors:** Christine J. Boinett, Simon R. Harris, Gemma C. Langridge, Elizabeth A. Trainor, Tod J. Merkel, Julian Parkhill

**Affiliations:** a Wellcome Trust Sanger Institute, Hinxton, Cambridgeshire, United Kingdom; b Division of Bacterial, Parasitic and Allergenic Products, Center for Biologics Evaluation and Research, FDA, Bethesda, Maryland, USA

## ERRATUM

Volume 3, no. 3, e00657-15, 2015. Page 1, column 1, line 25: “Megaruptur” should read “Megaruptor.”

